# CNV-seq, a new method to detect copy number variation using high-throughput sequencing

**DOI:** 10.1186/1471-2105-10-80

**Published:** 2009-03-06

**Authors:** Chao Xie, Martti T Tammi

**Affiliations:** 1 Department of Biological Sciences, National University of Singapore, Singapore; 2 Department of Biochemistry, National University of Singapore, Singapore; 3 Karolinska Institutet, Department of Microbiology, Tumor and Cell Biology, Stockholm, Sweden

## Abstract

**Background:**

DNA copy number variation (CNV) has been recognized as an important source of genetic variation. Array comparative genomic hybridization (aCGH) is commonly used for CNV detection, but the microarray platform has a number of inherent limitations.

**Results:**

Here, we describe a method to detect copy number variation using shotgun sequencing, CNV-seq. The method is based on a robust statistical model that describes the complete analysis procedure and allows the computation of essential confidence values for detection of CNV. Our results show that the number of reads, not the length of the reads is the key factor determining the resolution of detection. This favors the next-generation sequencing methods that rapidly produce large amount of short reads.

**Conclusion:**

Simulation of various sequencing methods with coverage between 0.1× to 8× show overall specificity between 91.7 – 99.9%, and sensitivity between 72.2 – 96.5%. We also show the results for assessment of CNV between two individual human genomes.

## Background

DNA copy number variation (CNV) has long been known as a source of genetic variation, but its importance has only been recognized recently [1,2]. In a landmark study in 2006, Redon and colleagues found that 1,447 CNV regions cover at least 12% of the human genome, with no large stretches exempt from CNV [3]. The CNV regions cover more nucleotide content per genome than single nucleotide polymorphisms (SNPs), suggesting the importance of CNV in genetic diversity [3]. A common way to detect CNV is to utilize microarray-based methods [4]. The most commonly used method, array comparative genomic hybridization (aCGH) was first used to detect CNV a decade ago [5,6].

Microarray-based methods have revolutionized the way of how large-scale genome studies are carried out. Today, the next-generation sequencing technologies are transforming biology research [7]. The rapid development of new sequencing technologies is continuously increasing the speed of sequencing and decreasing the cost. The next-generation sequencing, such as 454 [8], Solexa [9] and SOLiD [10] have already showed advantages over microarrays in several aspects. Apart from being rapid and cheap, data produced by sequencing can be re-used for varied purposes as opposed to data from microarray-based methods that can usually solely be used by one specific study. In addition, reproducibility has been one of the major challenges for microarray technology [11]. The once revolutionizing microarray-based ChIP-Chip technology is being replaced by ChIP-Seq, in which the DNA fragments are sequenced instead of being hybridized to an array [12]. Sequencing-based methods are also used to produce genome-wide DNA methylation profiles, detect SNP, study chromosome translocations and RNA transcriptome profiling [13-20].

Variation in sequencing coverage in genome assemblies has been used as an indicator for potential CNV between an assembled genome and shotgun data from another genome [21,22]. This is analogous to a comparison of copy number between microarray probes and a single set of DNA fragments. There are two major problems with this kind of approach. Given a certain hybridization condition, hybridization efficiency varies among microarray probes. Likewise, given a certain alignment threshold, sequencing errors in combination with differences between genomes may result in erroneous distribution of the reads.

Secondly, the number of probes on a microarray does not represent the real copy number of probe sequences in a genome. Likewise, the copy number of DNA segments in an assembled genome may not represent the true one. Notably, the regions containing multiple copies are the most difficult to assemble correctly and is still the key unsolved problem in shotgun assembly [23]. Assembly errors like these cause false variation in the sequencing coverage and thus yield erroneous indication of CNV.

In this paper we describe an efficient solution based on a robust model that combines the advantages of aCGH and high-throughput sequencing. We also assessed CNV between two individuals (Dr. J. Craig Venter [24], Dr. James Watson [21]). An implementation of our method is freely available at .

## Results and discussion

### The Model

**Figure 1:**
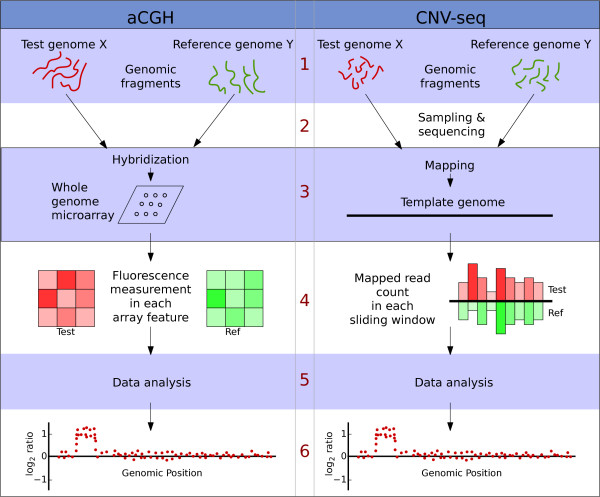
**A comparison of the conceptual steps in aCGH and CNV-seq methods**. 1. Starting material in both cases is genomic fragments from two genomes. 2. In CNV-seq the fragments are samples and sequenced. 3. Genomic fragments are directly hybridized on to an array. In CNV-seq the mapping is performed by sequence alignment. 4. In microarray the light intensities reflect the number of hybridized fragments. In CNV-seq the number of mapped reads are counted directly. 5. Data analysis, including estimation of copy number ratios, confidence values, etc. 6. Output of the results.

We have developed a method to detect CNV by shotgun sequencing, CNV-seq. The method is based on a robust statistical model that allows confidence assessment of observed copy number ratios and is conceptually derived from aCGH (Figure 1). The microarray-based procedure, aCGH involves a whole genome microarray where two sets of labeled genomic fragments are hybridized. Instead of a microarray, CNV-seq uses a sequence as a template and two sets of shotgun reads, one set from each target individual, *X *and *Y *(Figure 1). The two sets of shotgun reads are mapped by sequence alignment on a template genome. We use a sliding window approach to analyze the mapped regions and CNVs are detected by computing the number of reads for each individual in each of the windows, yielding ratios. These observed ratios are assessed by the computation of a probability of a random occurrence, given no copy number variation.

The random sampling in shotgun sequencing results in uneven coverage that may lead to observed coverage ratios that falsely imply CNV. Therefore, a statistical model is essential for the assessment of the probability of false positive ratios. The average number of reads in a region of a genome is dependent on the total number of reads sampled, the length of the genome and the length of the region. We use this relationship to compute a minimum sliding window size to achieve a desired minimum confidence level of the observations.

The mean number of reads for *X *and *Y *genomes in a sliding window determines the distribution of the ratios. The number of reads in a window is approximately distributed according to Poisson, Po(*λ*), where the mean number of reads per window is *λ*, given by

(1) $$\lambda =\frac{NW}{G}$$

where *N *is the total number of sequenced reads, *G *is the size of the genome and *W *is the size of the sliding window, and *W *< <*G*. We use the Gaussian distribution to approximate the Poisson distribution with mean and variance *λ *= *μ *= *σ*^2^. This approximation is good when the mean number of reads per window is greater than 10 with continuity correction.

**Figure 2:**
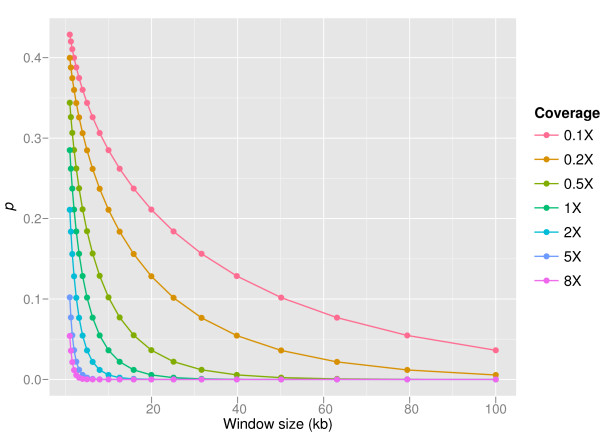
**Dependencies of *p *in CNV-seq**. The relation of *p *and sliding window size is shown on 0.1× to 8× sequence coverage for log_2_(*r'*) = 0.6 and average read length 250 bases. The values are computed using equation (5). Increased window length results in decreased probability, *p *of observing ratio *r' *or higher by cheer chance. It is possible to compensate lack of coverage by increasing the window size, but this results in lowering the resolution.

The predicted copy number ratio, *r *in each sliding window can be computed by

(2) $$r=z\times \frac{N_{Y}}{N_{X}}$$

where *z *is the ratio of read counts in the window and *N*_*X *_and *N*_*Y *_are the total number of reads in the genomes *X *and *Y *respectively. Assuming an independent distribution of the read counts, we can obtain a probability, *p *of a copy number ratio being *r *or divergent from 1:1 ratio by a random chance. For this purpose, we need the distribution of the read count ratio *z*. This distribution is given by Gaussian ratio distribution [25]. The computation of this distribution is cumbersome, but it can be transformed to another variable, *t*, by Geary-Hinkley transformation [26]:

(3) $$t=\frac{\mu_{Y}z-\mu_{X}}{\sqrt{\sigma_{Y}^{2}z^{2}+\sigma_{X}^{2}}}$$

where *μ*_*X*_, $\sigma_{X}^{2}$, *μ*_*Y *_and $\sigma_{Y}^{2}$ are the means and the variances for *X *and *Y *respectively. The new variable *t *approximately have a standard Gaussian distribution when the mean number of reads per window is greater than 6 in *Y *and less than 40,000 in *X*. The *p*-value can be computed by

(4) $$p=\{\begin{array}{ll} 2\times (1-\Phi (t)) & \text{if }r\geq 1,\\ 2\times \Phi (t) & \text{if }r<1. \end{array}$$

where Φ (*t*) is the cumulative standard Gaussian distribution function. The probability *p *decreases with increasing sliding window size (Figure 2) and we would like *p *to be as low as possible. Conversely, increased sliding window size leads to a decreased resolution of CNV regions. Therefore it is advantageous to compute a window size, which yields the best possible resolution according to a preset threshold of *p *for *r*. Based on the above equations, We can calculate the best possible resolution, or the theoretical minimum window size, *W *by

(5) $$W=\frac{(N_{X}{r^{\prime }}^{2}+N_{Y})GT^{2}}{{\left(1-r^{\prime }\right)}^{2}N_{X}N_{Y}}$$

and

(6) $$T=\{\begin{array}{ll} \Phi^{-1}(0.5\times (1-p')) & \text{for }\det\text{ecting CNV with ratio}\geq r^{\prime },\\ \Phi^{-1}(0.5\times p') & \text{for }\det\text{ecting CNV with ratio}\leq r^{\prime }. \end{array}$$

where *p' *is the desired significance level, and *r' *is the CNV detection threshold ratio. Φ^-1 ^is the inverse function of Φ. The number of reads sampled will affect the minimum window size. For example, if one wants to detect CNV with ratio ≥ 3 : 2 at significance level 0.002, a genome size of 3 G bases and 10 M reads in both genomes will yield the minimum window size of 37,243 bases, while 1 M reads will yield the window size of 372,431 bases. The use larger number of reads allows detection of ten times shorter CNV.

An alternative approach is to calculate the range of copy number ratios that can be detected at a certain significance level *p'*, with a predefined window size *W'*:

(7) $$r\geq \frac{1+\sqrt{1-AB}}{B},\text{ and},\;r\leq \frac{1-\sqrt{1-AB}}{B}$$

where

(8) $$A=1-\frac{{\left(\Phi^{-1}(1-0.5\times p')\right)}^{2}G}{N_{X}W^{\prime }}$$

and

(9) $$B=1-\frac{{\left(\Phi^{-1}(1-0.5\times p')\right)}^{2}G}{N_{Y}W'}$$

### Validation

In order to assess the performance of CNV-seq, we used simulated and real human data. For the simulation of shotgun data, in total of 101 genomes were constructed, containing varied number, sizes and locations of CNV regions, SNP and short insertions/deletions (indels). We simulated three sequencing methods, Solexa, 454 and Sanger [27] for each constructed genome on 0.1× to 8× coverage. This resulted in the total of 8,400 simulations.

**Figure 3:**
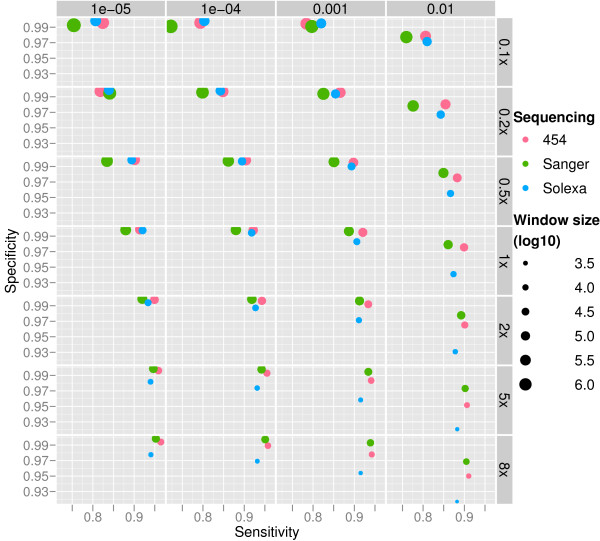
**Performance of CNV-seq**. The performance of CNV-seq on data simulating 454, Sanger and Solexa methods. Results are shown for 0.1×–8× coverages (right) and *p*-value range of 10^-5^-10^-2 ^(top). Each dot represents an average of 100 simulations and the size of the dots represents the window (log_10_) size, i.e. resolution used. The window sizes are calculated using equation (5).

**Figure 4:**
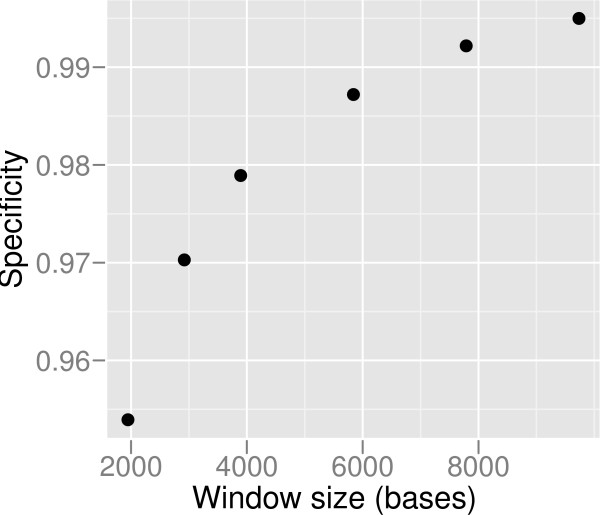
**Specificity vs window size**. In order to increase specificity, a larger than the theoretical minimum window size can be used by sacrificing resolution. The specificities using 1×, 1.5×, 2×, 3×, 4×, and 5× of the theoretical minimum window size are shown, for simulated Solexa sequencing data at 8× coverage.

The Figure 3 shows the results of the simulations on varied coverage and varied *p' *for constant log_2_(*r'*) = 0.6. Each dot represents an average of 100 simulations and the sizes of the dots reflect the sizes of the lengths of the sliding windows that are the theoretical minimum lengths, given by equation (5). The overall specificity for our method is between 91.7 – 99.9%, the sensitivity between 72.2 – 96.5% with the median of 99.4% and 89.9% respectively. The mean sequence length is dependent on the technology simulated. Thus, in order to reach the same coverage, a larger number of fragments need to be sequenced when sequencing is performed with Solexa, which produces short reads compared to the Sanger and 454 methods. According to our model, the largest number of sequenced reads yields the shortest length of the sliding window and thus the best resolution. The range of window sizes in our simulations varies from 1,103 bases to 2,951,792 bases, decreasing with increasing average sequencing coverage. The results show that our model performs well in the presence of errors. Despite of increased resolution due to shortening of the sliding window size, the sensitivity is increased together with increased sequencing coverage. Slight drop in specificity with increasing sequencing coverage can be observed (Figure 3). This is likely to be due to SNPs, short indels, and read mapping errors, that are not considered in our statistical model and have a more profound effect on small windows. The specificity does not drop in error free data. The effect of errors may be reduced by using a window size that is larger than the theoretical minimum. For example, the theoretical minimum window for 8× Solexa sequencing at *p *= 0.001 is 1947 bases. This window size gives a specificity of 95.4%, while a 2 times larger window yields specificity of 97.8% (Figure 4).

### Analysis of human data

**Figure 5:**
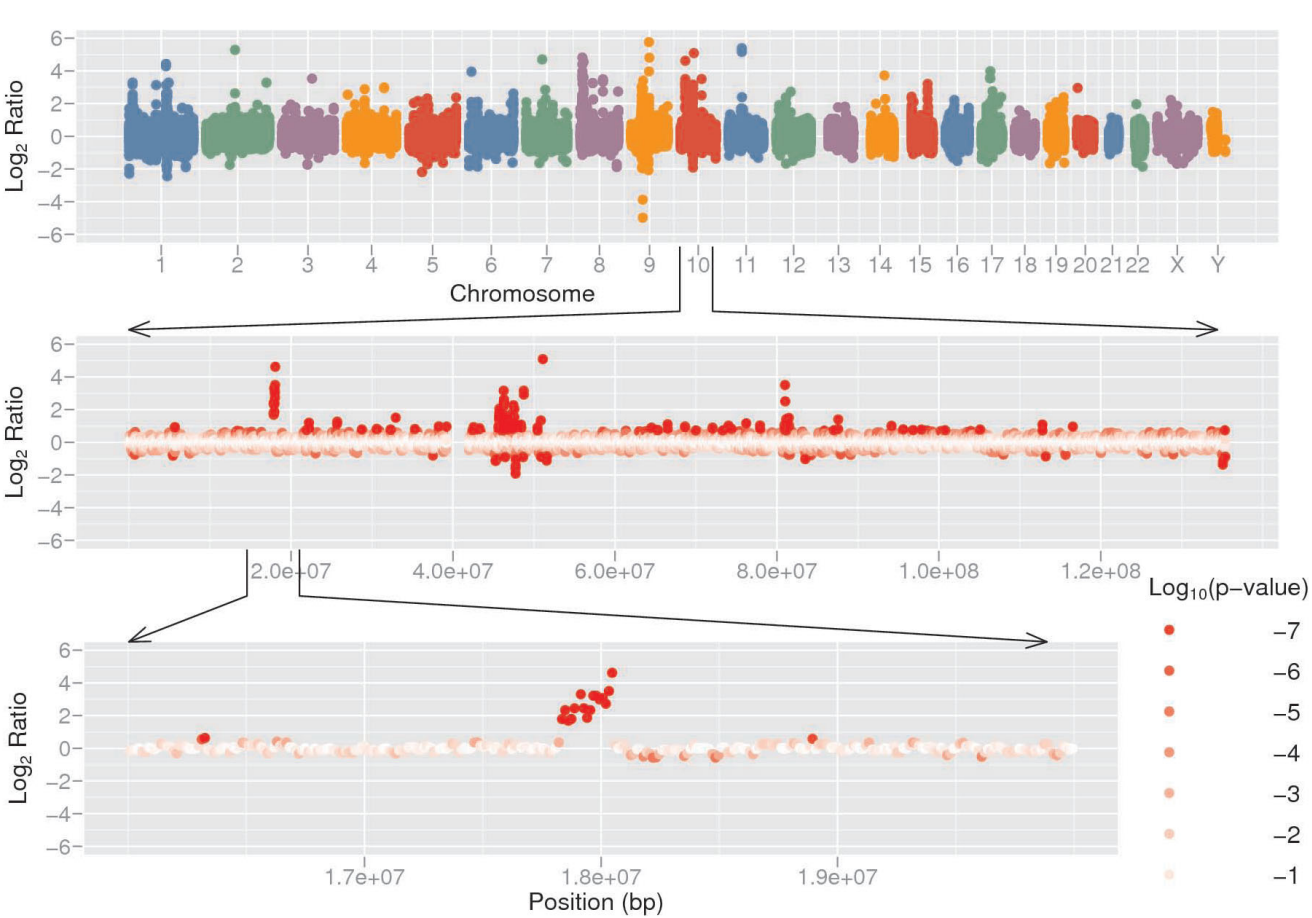
**Copy number variation between two human individuals**. Copy number variation detected by CNV-seq using shotgun sequence data from two individuals, Venter and Watson. The top panel shows a genome level *log*2 ratio plot. The middle panel shows the plot for chromosome 10. The bottom panel shows detailed view of a CNV region in chromosome 10. The red color gradient in the middle and bottom sections represents log_10 _*p *calculated on each of ratios.

The genomes of two individuals, Dr. Craig J. Venter and Dr. J. Watson were recently sequenced on 7.5× and 7.4× coverage respectively [21,24]. The genome of Dr. Craig J. Venter is sequenced using Sanger method and Dr. J. Watson's genome using 454 technology. We compared the two genomes using CNV-seq (Figure 5 and Additional File 1). The thresholds *p' *= 10^-5 ^and log_2_(*r'*) = 0.6 yield sliding window size of 26,481 bases for autosomal chromosomes. The sex chromosomes have a lower sequencing coverage than autosomal chromosomes, therefore larger window sizes are used: 72,044 bases for chromosome X and 269,032 bases for chromosome Y. We identified 174 contiguous regions covered by four or more consecutive windows. The sizes of these regions range from 66,202 bases to 941,612 bases.

**Figure 6:**
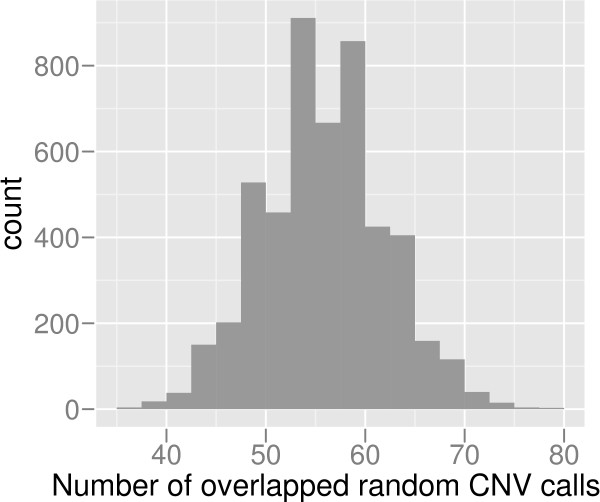
**Permutation test of CNV calls**. A permutation test was performed in order to test the significance of our CNV calls. The histogram shows the number of CNV calls overlapping with CNV in DGV. The X-axis shows the number of calls overlapping with DGV. The Y-axis shows the frequency of the overlapping number in 5,000 sets of permuted CNV calls.

The comparison of the 174 CNV calls with those in the Database of Genomic Variants (DGV) [2] revealed 142 of the CNV calls to overlap more than 50% with previously reported CNV regions. In order to asses the significance of CNV calls, we performed 5,000 permutation tests, using 174 randomly distributed CNV regions of the same sizes as in the original experiment. In average, only 56 and maximum 78 of 174 regions overlap more than 50% with CNV in DGV (Figure 6) 5,000 random sets. The real CNV calls have significantly larger overlap with DGV (*p *= 0).

We also intersected the CNV calls with the CNVs identified by aCGH in the two genomes. There are 23 and 45 CNV regions reported in Watson's and Venter's genome respectively [21,24]. We found 15 of our CNV calls overlap with 10 of previously reported Watson's CNV regions, and only 11 of our CNV calls overlap with 5 of Venter's. The low overlap with Venter's CNV calls made by aCGH is not surprising, for the reason that the majority of the CNV regions were detected by only one of three microarray platforms [24]. There are 121 CNV calls that made by CNV-seq but not aCGH and overlap with DGV data, suggesting that CNV-seq can detect CNV regions that were missed by aCGH. One of these regions is shown in Figure 5 (bottom panel), a 238 kb region (copy number ratio 6:1, *p *= 0) containing two genes (FAM23B, MRC1L1) and one miRNA (hsa-mir-511-2). We have used stringent thresholds in our analysis, thus by lowering thresholds, such as p-value and the number of consecutive windows, will increase the number of reported CNV calls.

A major assumption in CNV-seq is that shotgun sampling of DNA fragments is random, and therefore the CNV calls made by CNV-seq are not due to different sequencing bias between the two sets of data compared. When the two sets of data are prepared in the same way, this assumption is valid. However, when the shotgun sequences are generated using two different sequencing methods, the assumption may not hold any more. Solexa sequencing reads are recently reported to be GC-biased dependent on a library preparation procedure [28]. Venter's genome was sequenced using 454 and Watson's genome was sequenced using the Sanger method. We compared the distribution of GC frequencies in the shotgun reads in both genomes. There are no significant differences between the two distributions (*p *= 0.2106, Kolmogorov-Smimrov test).

## Conclusion

We have developed a method to detect CNV using shotgun data. Our approach not only combines the advantages of microarray methods and high-throughput sequencing, but is also based on a robust statistical model allowing confidence assessment. We tested the approach on both simulated and real data and the results show that the method can be applied to relatively low sequencing coverage with good specificity and sensitivity. We have also developed a model to compute the theoretical limit of resolution for given data at a desired confidence level.

We expect the continued rapid development of sequencing technologies to further lower the cost and increase the speed of sequencing. Thus, sequencing-based approaches are likely to gain increased advantage over microarrays. Next-generation sequencing methods mostly produce a large number of short reads and our results show that the number of reads sequenced – not the length of the reads, is the most important factor that determines the resolution, i.e. larger number of sequenced fragments results in increased resolution. Alternatively, given a constant resolution an increase in the number of sequenced reads will result in increased sensitivity and specificity. Therefore, a large number of short reads is an advantage as opposed to a small number of long reads.

## Methods

### Simulations

The human chromosome 1 (NCBI build 36) was used to construct one diploid reference genome and 100 diploid test genomes. The unmodified chromosome 1 sequence was used as the template genome. The test individual genomes are constructed by the introducing CNV, SNPs and short indels. The CNV is introduced into each of the test genomes by concatenating the two chromosomes and by selecting nine source sequences at random positions to replace 26 target sequences at random positions. Four of the nine source sequences are used four times each to replace four random target sequences and the remaining five of the nine sequences are used to replace two random target sequences each. The procedure results in the total of 35 segments in each of the 100 simulated test genomes with the following copy number ratios: 26 with ratio 1:2, five with ratio 4:2 and four with ratio 6:2. The length of the source sequences is 10^*k*^, where *k *is a random number between log_10 _500 and log_10 _2 M, yielding the median length of 26,464 bases and the mean 234,065.7 bases. In addition, each test genome is modified by randomly introducing 5 SNPs/kb and short, 1–3 bp insertions/deletions with the frequency of 0.5 indels/kb.

The reference genome is constructed the same way as the individual test genomes, except no CNV was introduced.

We simulated the shotgun sequencing process for test and reference genomes by using real sequence quality files, specific for each sequencing method. The quality files used for Sanger and 454 sequencing were downloaded from the personal genome projects of Venter [24] and Watson [21] in Trace Archive [29], respectively. For the simulation of Solexa method we used quality files from the project SRA000261 in Trace Archive. The lengths of the quality files define the read lengths at a random starting position. The errors were introduced according to quality values given in the quality files. Both Sanger and 454 methods use Phred quality values [30], *q *and the error probabilities, *e *are given by *e *= 10^*q*/-10^. The errors are introduced by generating a random number *R *between 0 and 1. If *R *<*e*, then one of the following errors will be introduced: Substitution to one of the three remaining bases, an insertion or a deletion. The probability of an indel is 10% of all introduced errors with the equal ratio of indels. The base frequency in the source genome is used to calculate the frequency of each base, which is in turn used to give the insertion and substitution probability. The Solexa quality values, *q*_*s *_can be converted to Phred quality scores as follows

(10) $$q=10\times \log_{10}\left(1+10^{\frac{q_{s}}{10}}\right)$$

We simulated the shotgun process for 0.1×, 0.2×, 0.5×, 1×, 2×, 5× and 8× coverages.

The performance is measured by counting the number of sliding windows giving a correct alternatively an incorrect prediction. Our model describes the theoretical limit of detection for given data with given *r' *and *p'*. The true copy number ratio of each window is known in the simulated data, i.e. the true *r*. All windows where true *r *≥ *r' *or *r *≤ 1/*r' *should be classified as CNV in order to achieve 100% sensitivity. Similarly, all windows where true *r *≤ *r' *or *r *≥ 1/*r' *should not be classified as CNV in order to achieve 100% specificity.

### CNV detection in human data

The shotgun sequencing data were downloaded from the personal genome projects of Venter and Watson in Trace Archive. The template genome was downloaded from Ensembl [31], human genome assembly, NCBI Build 36. The thresholds *p' *= 10^-5 ^and log_2_(*r'*) = 0.6 are used. Given the data these thresholds yield the window size, *W *= 26, 481 bases for autosomal chromosomes, 72,044 bases for chromosome X and 269,032 bases for chromosome Y.

### CNV-seq

All calculations are performed using R [32] and sequences aligned by BLAT [33]. The whole procedure is automated by Perl  scripts.

## Authors' contributions

CX and MT contributed to all aspects of this research. Both authors read and approved the final manuscript.

## Supplementary Material

Additional File 1 **CNV regions identified between Venter's and Watson's genomes.** The 174 identified CNV regions, including the length, location, *log*2 ratio, and *p*-value for each of the regions. Click here for file
